# A systems analysis of ward rounds in plastic surgery at a single center

**DOI:** 10.1097/IJ9.0000000000000018

**Published:** 2017-06-22

**Authors:** Riaz Agha

**Affiliations:** Department of Plastic Surgery, Stoke Mandeville Hospital, Buckinghamshire Healthcare NHS Trust and Kellogg College, University of Oxford, Oxford, UK

**Keywords:** Ward round, Plastic surgery, Quality improvement, Systems analysis, Interviews, Qualitative analysis

## Abstract

Ward rounds permeate health care delivery worldwide and form an important daily activity within all hospitals. In this study, the daily morning ward round in plastic surgery was examined from a teleological and systems point of view.

**Methods:**

Data were gathered from the following sources to inform the systems analysis: patient interviews, staff interviews, direct observations of the ward round on multiple occasions, and through process mapping.

**Results:**

To better understand the ward and its layout, a schematic of it was developed. Following observations of the ward round and the way in which decisions are made on it, an IDEF0 map was developed. Three patients were invited to take part in the study. Three members of staff were interviewed. The overarching themes from the interviews related to aspects of communication which this paper details.

**Conclusions:**

This small-scale study demonstrates how the ward round as a process can be assessed, including product quality, process quality, the measurement and management of capacity, the role of standardization, the role and significance of bottlenecks, the key information flows, including the role of feedback, and the motivation and incentives of system participants and ideas for improvement generated.

## Abstract 2

Supplemental Digital Content is available in the text.

## What is a ward round and why is it important

Ward rounds permeate health care delivery worldwide and form an important daily activity within all hospitals^1^. The Oxford English dictionary^2^ defines a ward round as:

visits paid by a doctor in a hospital to each of the patients in their care or in a particular ward or wards

But ward rounds are of course more complicated than this, involving more than just a “visit” and by usually a team of professionals, not just a lone doctor.

Ward rounds are considered essential for communicating with patients and their relatives, involving patients in their own care, monitoring progress and arranging investigations, an integrated management plan and coordinating discharge^3^. However, they are a complex task requiring a team of professionals (doctors, nurses, and allied health professionals), medical knowledge, patient-specific knowledge, communication skills, clinical skills, patient management and teamwork skills, and their integration with data from a variety of sources (the patient themselves, bedside observation charts, pathology reports, etc).

## Problems with ward rounds—a brief overview

O’hare in his paper on the *Anatomy of the ward round*, described them from the patients perspective as:

a passing parade of white coats that arrive at the bedside unannounced, speaks, listens (occasionally), and murmurs in jargon only to pass on all too quickly.

Ward rounds have come into sharper focus recently with the Royal Colleges of Physicians and Royal Colleges of Nursing stating that ward rounds are being neglected and that they needed to be reprioritized to become a “cornerstone” of daily life in hospitals again. New joint guidance from the Royal Colleges of Physicians and Royal Colleges of Nursing cites “considerable variability in the way ward rounds are conducted” and that they need to become multidisciplinary to be effective^4^.

## Ward rounds in plastic surgery

The ward round is of vital importance in the specialty of plastic surgery. UK-based Consultant Plastic Surgeon Jeremy Rawlins^5^ has written about the pivotal role of the ward round and the challenges associated with it. Rawlins discussed the combination of communication and management skills needed for a successful ward round and highlighted some principles for managing the transition from “junior member of the ward round to the person charged with leading it.” These are listed below:

1. Know the team
2. Take charge
3. Say something
4. Use everybody
5. Listen
6. Bring people in
7. Be alert to psychosocial needs
8. Teach them a thing or two
9. Be systematic but be flexible
10. Be diplomatic
11. Have a plan
12. Summarize
13. Reflect

## Objective

In this study, the daily morning ward round in plastic surgery was examined from a teleological and systems point of view. This paper has been reported in line with the standards for reporting qualitative research: a synthesis of recommendations (SRQR) reporting guidelines^6^.

## Methods

### Design

Data were gathered to inform the systems analysis from a number of sources including: patient interviews, staff interviews, direct observations of the ward round on multiple occasions, and through process mapping.

### Setting

**Figure 1:**
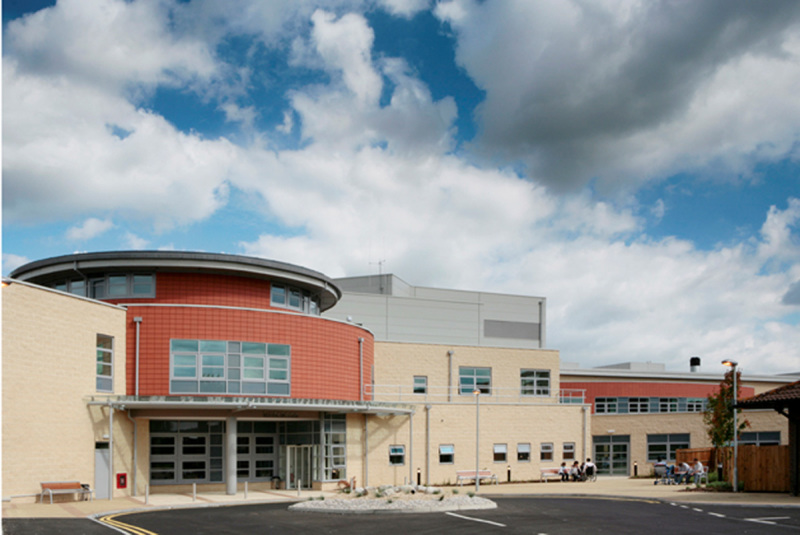
Main entrance to Stoke Mandeville Hospital.

Stoke Mandeville Hospital (SMH) is part of Buckinghamshire Healthcare NHS Trust along with Wickham and Amersham hospitals. The hospital is located on the edge of the market town of Aylesbury in Buckinghamshire. The hospital has 479 beds and treats over 48,000 inpatients and 219,000 outpatients a year^7^. The hospital is known for its spinal injuries unit as well as being the birthplace of the Paralympic movement. In addition, the hospital provides a 24-hour accident and emergency service, maternity, cancer care and provides specialist services like plastic surgery and a regional burns unit (**Fig.** **1**).

### The plastic surgical service

Plastic surgery at SMH provides an on-call and elective service for hand surgery, burns, breast surgery, congenital deformities (including hypospadias), skin cancer, and wound management. Patients are admitted to ward 16 where they share 23 beds (including 3 side rooms) with gynecology and general surgical patients. Pediatric patients are admitted to ward 3 and adult burn patients to the dedicated burns unit. For the purposes of this study, only the daily morning ward round on ward 16 was studied, where the majority of patients are.

### Data collection and participants

#### Personal observations

For 6 days within a 2-week period in November 2012, the daily morning ward round was observed by the author. The ward round typically starts at 8:30 to 8:45 after the morning trauma meeting. As problems arose notes were made on a patient list being carried by the observer. The ward round was timed and the number of patients seen noted. A problem was defined as anything which:

1. “Burns energy off from the organization or its staff” or leads to their frustration.
2. Leads to increased “cultural entropy” in the system^8^. Richard Barrett defined cultural entropy as:
  the amount of energy in an organisation that is consumed in unproductive work. It is a measure of the conflict, friction and frustration that exists within an organization^9^.

#### Patient and staff interviews—selection of participants

In November 2012, over 3 consecutive days, patients were offered a chance to participate in the study. Patients were selected if they had been seen on at least 2 ward rounds. Day case admissions and 23-hour stay patients were excluded. Patients with dementia who would be unable to comprehend the consent form or the questions were excluded.

#### Patient and staff interviews—a grounded theory approach

Patients and staff were interviewed using a grounded theory approach as espoused by Glaser and Strauss^10^. Such an approach respects the participants subjective interpretation of their experiences and the social processes within their professional socialism^11^. This philosophy encourages the researcher to view patients or research participants as being interactive components of their environment and understand that human phenomena require acknowledgment that people take meaning from experiences shared with others^12^,^13^. This approach also allows the interviewer to explore emerging themes during the discussion and would prevent it from being framed too narrowly^14^.

The interview would begin each time with the same open question and then subsequent questions would follow dynamically based on the answer to the first and the issues raised. The opening question was:

What are your thoughts and feelings about the daily morning ward round.

### Ethical considerations

Following discussion with the Stoke Mandeville Hospital Research and Development department and the Clinical Audit department as well as consultation of relevant National Research Ethics Service (NRES) guidance^15^, this work was defined as a “service evaluation” rather than research or audit. As a result ethical or institutional review board approval was not required or sought.

All patients who participated in the study through face-to-face interviews, did so through an informed consent process. A consent form was developed (Appendix 1, Supplemental Digital Content 1, http://links.lww.com/IJSO/A2) based on the World Health Organisation Research Ethics Review Committee template consent form for qualitative studies^16^. Interviews were recorded either in written and/or audio format and patients specifically had to opt-in for audio recording. Audio recording was done using an iPhone 4, which was password protected with the auto-lock feature activated. It had the advantage of being always available and provided good quality audio. Once synchronized with a password protected and encrypted Apple Macbook air computer, the audio recordings were deleted from the iPhone.

Staff interviews were either done through audio recording or the interview was written down. In the case of the latter, the staff member was then asked to read and check the accuracy of what had been transcribed by the interviewer and then to sign the statement.

### Data Analysis

All audio recordings were transcribed into a Microsoft Word 2011 document and playback was repeated following transcription to ensure fidelity. Transcripts were studied line by line and a thematic analysis performed. Issues in relation to the ward round were identified and logged.

## RESULTS

**Figure 2:**
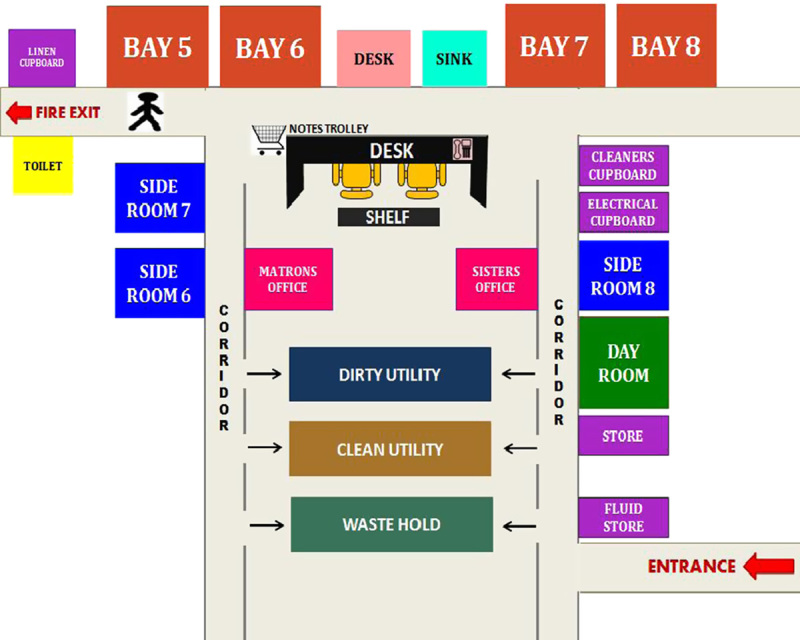
A schematic of ward 16 (not drawn to scale). The ward round typically starts in bay 5 and finishes with the side rooms. Each bay contains 5 beds and a bathroom. Each side room contains an en-suite toilet. Side rooms 1 to 5 and bays 1 to 4 are located on an adjacent medical ward (which would be at the bottom of the schematic) not shown here.

To better understand the ward and its layout, a schematic of it was developed (**Fig.** **2**).

**Figure 3:**
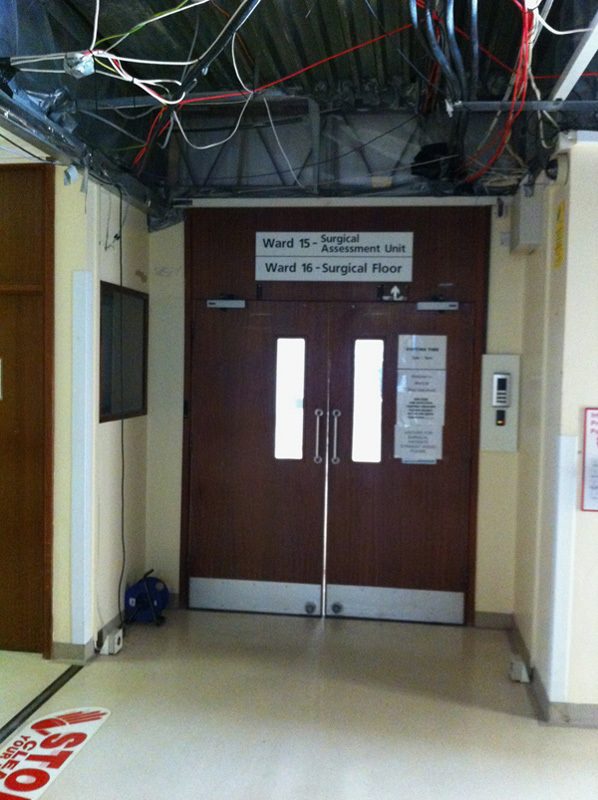
Photograph of the entrance to ward 16. The electrical cabling overhead was noted.

**Figure 4:**
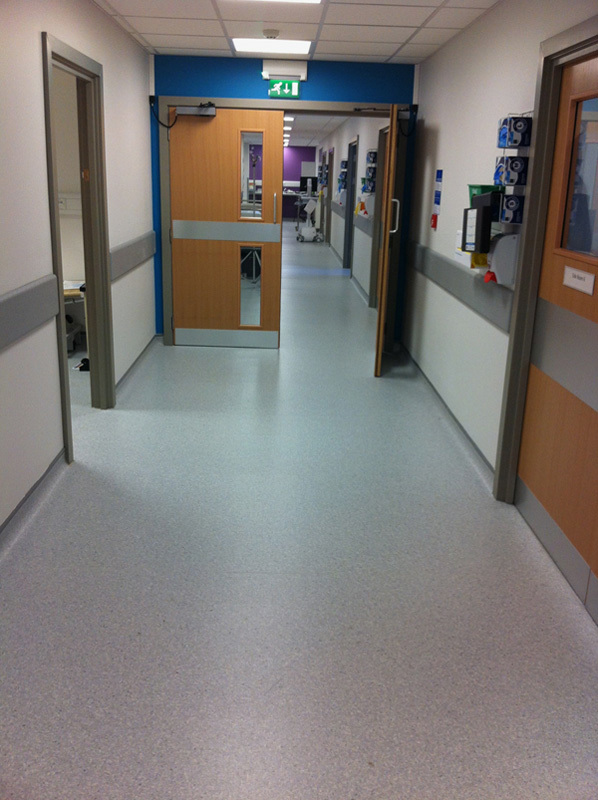
Photograph of a corridor on ward 16.

**Figure 5:**
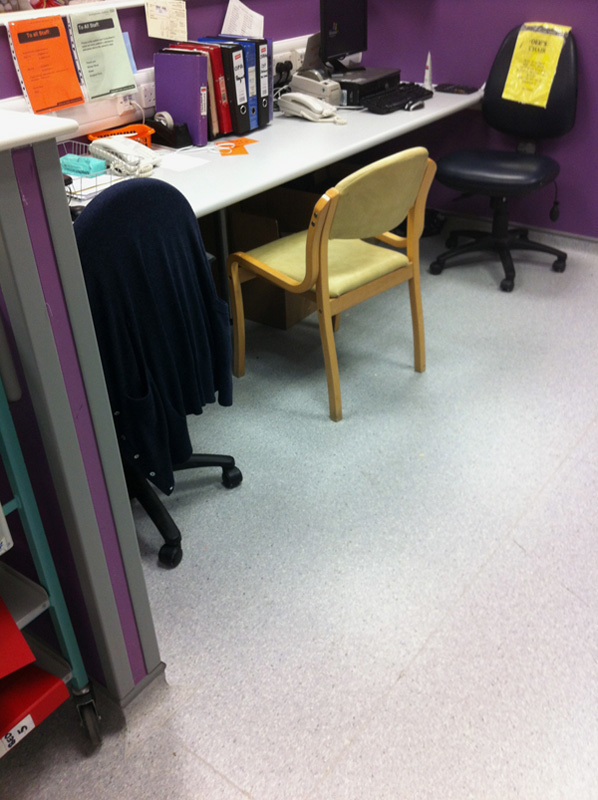
Photograph of the desk space on ward 16 in the morning. Two computers and 2 telephones but no printer, meaning the house officer has to go to the adjoining medical ward to print the patient list, as a result its often not done in time for the ward round.

**Figure 6:**
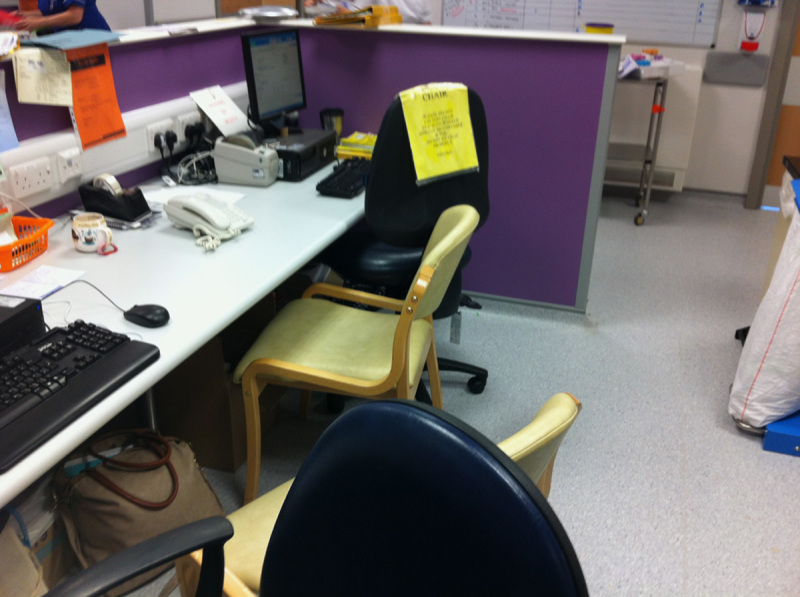
Photograph of the same desk later in the day, one of the telephones had been moved and an extra chair had appeared when only 3 people can fit into the desk space—an example of entropy in the system.

**Figure 7:**
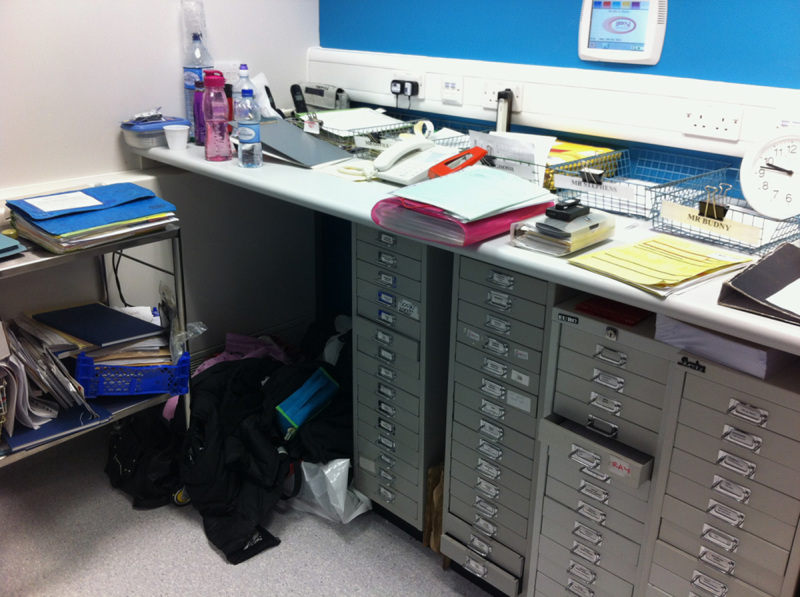
Photograph of the shelf space on ward 16. Very cluttered with little spare desk space to utilize.

**Figure 8:**
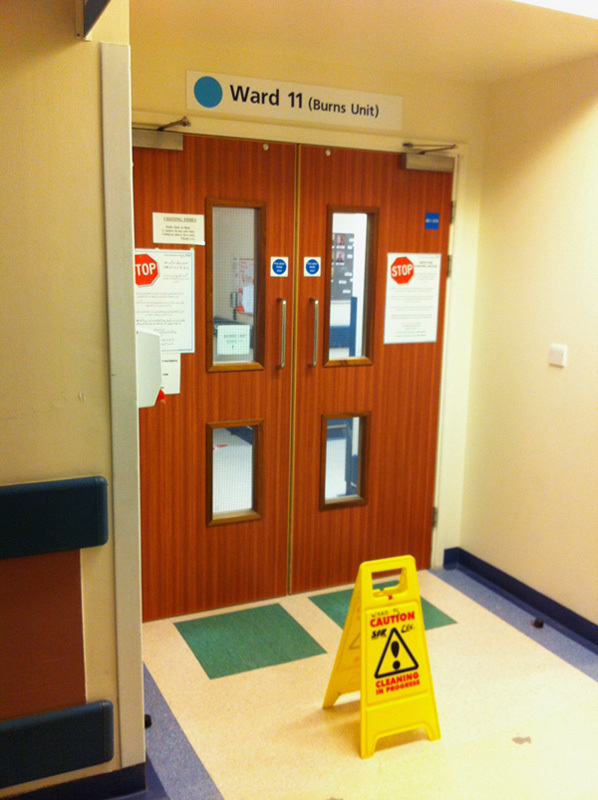
Photograph of the entrance to the Burns Unit. The Burns unit was not part of this study but a safety hazard in the form of a poorly placed “caution wet floor” sign was noted.

Some photographs of the ward are shown (**Figs.** **3–8**).

In general, plastic surgery has between 5 and 12 patients on ward 16. These are a mix of trauma patients admitted via A&E and those admitted directly to the ward for elective procedures.

### Personal observations

The ward round typically consisted of the following staff members:

- One Specialist Registrar (a middle grade doctor with 6 to 11 y postgraduate experience).
- One to 3 Senior House Officers or SHOs (junior doctors with 2 to 4 years of postgraduate experience).
- A House Officer (recent graduate from medical school and started work in August 2012).
- One nurse.
- One or 2 physiotherapists/occupational therapists.

The results of the observations are shown in **Table** **1**.

**Table 1:**
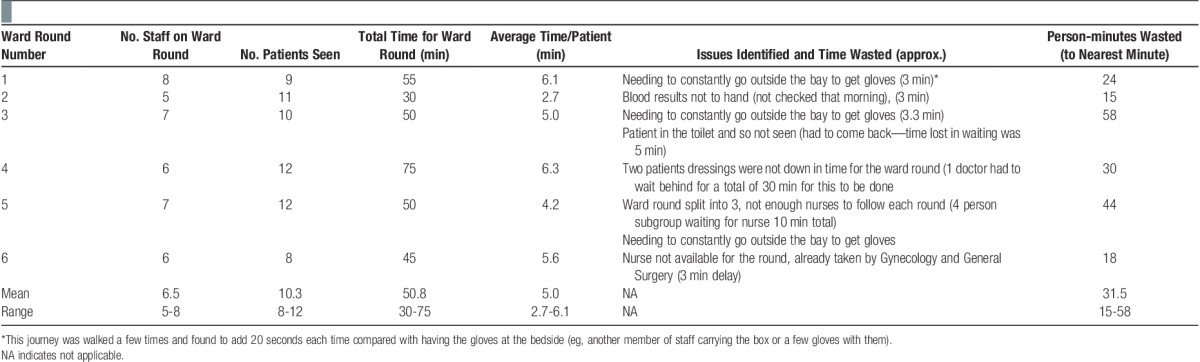
Observations from the ward round.

On all 6 days, all plastic surgical patients on ward 16 were seen with no omissions. All requested tests from the ward round were ordered by the house officer after the ward round had finished, there was no delay for this during the ward round itself for this activity. A patient list was not ready in time for the start of the ward round on any of these days. It was provided at the end of the ward round after the house officer had gone to another ward to print it.

### Interviews

Three patients were invited to take part in the study. All 3 consented to take part and to have the interview audio recorded. Six patients on the ward were excluded for being days cases or not having been seen on 2 or more occasions during the study period. One patient was excluded due to dementia. Three members of staff were interviewed (1 band 5 and 1 band 6 nurse and 1 house officer), 1 was audio recorded and the other 2 were written down.

The main themes emerging from the interviews are listed below, mostly they are centered on aspects of communication:

- Ward round is too quick and impersonal.
- Lack of privacy, feels intimidating.
- Too many people on the ward round.
- Don’t feel they can ask questions, feel “stupid.”
- Not sure who the members of the ward round are, do they belong to their consultant’s team?
- Not clear on the treatment plan.
- Use of technical jargon.
- Talking as if the patient is not there.
- Hearing different or conflicting information from different registrars.
- Doctors don’t know me well enough and may make a wrong decision.
- Miscommunication meant patient was fed and missed their operation slot.
- Would appreciate someone double checking that the patient is clear on the plan before they leave.

Portions of interviews are shown below (the full transcripts are provided in the Appendix, Supplemental Digital Content 1, http://links.lww.com/IJSO/A2):

#### Patient A

So what are your thoughts and feelings about the ward round that we do in the mornings?

It’s okay, I just feel sometimes it’s a bit too fast and a bit intimidating and there’s too many people there. But then I tend to ask my nurse, what’s going on once you’ve all lot have left so …. just spending an extra five minutes with the patient to make sure that the patient is aware of exactly what is going on …. I don’t think we are spoken to relevantly in that amount of time. So like if we have questions, they don’t feel, I don’t feel comfortable of being not able to ask in front of everybody what I want to know because I feel stupid … … I don’t know, it’s just like when everybody is on top of you, looking at you and wanting to see what’s going on its like.

And are you clear when we leave about what’s taking place, what the plan is?

No, not at all half the time. When things are being said that we need to do this, this and this we don’t always understand what you mean because it’s all technical and I have noticed that the consultant would talk to the other members of the staff rather than the patient of what being spoken over. So then I wait until a nurse comes in and then ask the nurse what’s going on.

#### Patient B

I think the confusion then comes, if the big round comes and then you get, I had a stray registrar come and tell me something different and I actually did question who he was in relation to the ward round …… Better introduction as to who they are perhaps …. I was thinking it’s quite nice if the junior doctor then comes back and double checks with the patient.

#### Patient C

It is very daunting, I mean you’ve got lots of different doctors at the end of your bed obviously when you are not feeling well anyway. But it’s very confusing when you don’t know what team that they’re working for. Years ago it used to be just your consultant and their team that was at the end of the bed. Now everybody’s plastics teams standing at the end of the bed with me and that is really quite daunting because you don’t remember everyone of who they are so you get a bit confused who, who is taking your care on.

#### Staff A—nurse band 6

Too many people on it. There should just be: one Registrar, one SHO (preferably the on-call one), house officer, physio/OT. Should not split up this can lead to chaos. A Consultant should be on the ward round when there is bed pressure as otherwise there is a four hour gap waiting for a Consultant who may or may not be in the hospital.

#### Staff B—nurse band 5

I found that some patients actually find it overwhelming because all doctors are stood around looking at them specially ladies breast surgery and it’s quite a personal thing to them to then have sort of eight or nine doctors all looking at them. They don’t like it. Also I find that sometimes two doctors look after that patient then two would go there and the nurse in charge trying to find where these three different break of doctors are all trying to say at the same time.

#### Staff C—house officer

Not enough computers. Can’t update the list. Ward is too small and you can’t fit the trolley and doctors into a bay. Patients are close together, no privacy. Amount of direction you get depends on the Registrar. You have to call the Registrar back to get discharge details like antibiotics and follow-up. One registrar in particular tells patients their fingers will fall off. This never goes down well and he upsets a lot of patients.

### Process mapping

**Figure 9:**
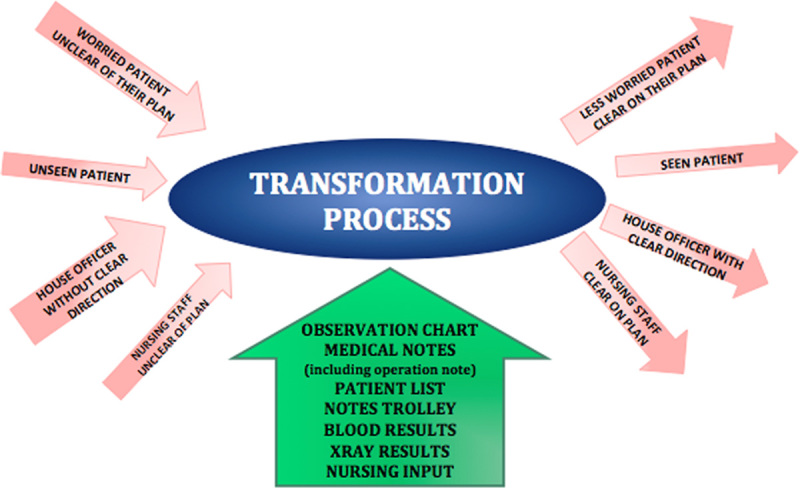
An IDEF0 map of the ward round.

Following observations of the ward round and the way in which decisions are made on it, an IDEF0 map was developed (**Figs.** **2**, **9**).

## Part A

### The definition of product and process quality that applies to the system

The goals of the daily ward round include:

1. Enhancing the quality of care
2. Improving multiprofessional communication
3. Addressing patient concerns and problems
4. Planning and evaluating treatment
5. Planning investigations (eg, x-rays and blood tests)
6. Planning discharge
7. Multiprofessional training and education

As a result one can state that product and process quality encompass numerous elements as highlighted below.

#### Product quality

The quality of the ward round as a product has a number of dimensions but as a starting point, reference was made to Lord Darzi’s report *High Quality Care for All*^17^, which emphasized clinical effectiveness, patient safety, and patient experience as being the key constituents of high quality in health care:

- Good clinical decision making—encompassing patient safety and clinical effectiveness.
- Patient experience—patient satisfaction with the experience of the ward round, questions answered, etc.
- Timely decision making.
- All patients are seen.
- Clear plans are articulated to the patient and other members of the ward round (junior doctors, nursing staff, physiotherapists, and occupational therapist) and documented in the medical notes.

#### Process quality

**Figure 10:**
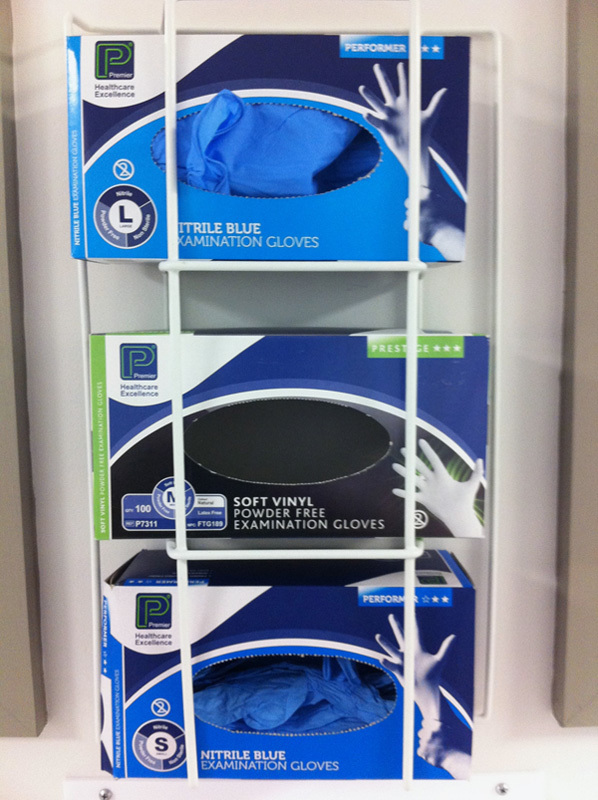
Photograph of glove boxes on ward 16. The stock of medium-sized gloves was depleted. As a result the wrong size was being used, making the examination more difficult technically and more uncomfortable for the examiner. The nonavailability of proper-sized gloves and the ensuing frustration may even lead to gloves not being worn at all, compromising infection-control policies.

**Figure 11:**
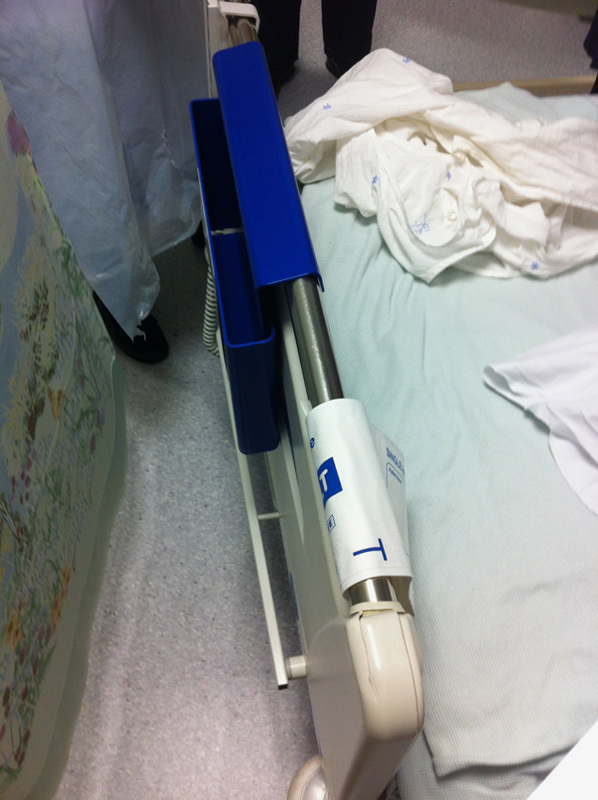
Photograph of a patient’s bed. No alcohol foam dispenser at the end of the bed making it less likely that hand hygiene will be performed between patients again compromising infection-control policies.

- All patient’s seen.
- Minimize missing information, duplication, wasted movements.
- Timely decision making.
- Availability of tools for the job, for example, medium-size gloves and alcohol foam dispenser (**Figs.** **10**, **11**).

### The measurement and management of capacity

Capacity on the ward traditionally relates to the number of beds available. In the context of this exercise, we also need to consider the time it takes to do the ward round and to see each patient. **Table** **1** shows the following range and means for such activity:

- Time to see a single patient varies from 2.7 to 6.1 minutes, with a mean time of 5.0 minutes.
- Total ward round time varies from 30 to 75 minutes, with a mean time of 50.8 minutes.
- The number of patients seen varied from 8 to 12, with a mean of 10.3.
- A high degree of variability was also shown for the number of staff on the ward round and the number of person-minutes wasted (**Table** **1**).

### The role of standardization

A significant issue found during ward rounds was the lack of standardization. Each day a different specialist registrar would do the morning ward round. This was a feature of both the staff and patient interviews.

#### Patient B

I think the confusion then comes, if the big round comes and then you get, I had a stray registrar come and tell me something different and I actually did question who he was in relation to the ward round.

#### Patient C

It is very daunting, I mean you’ve got lots of different doctors at the end of your bed obviously when you are not feeling well anyway. But it’s very confusing when you don’t know what team that they’re working for.

#### Staff A

Not necessary for every dressing to go down all the time for each Registrar who comes along. There should be one Registrar doing the ward round for the week so they know the patients, know the plan, etc.

As patients are mixed with gynecology and general surgery patients, it would conceivably be better to have all the plastic surgical patients in their own bays. This would make the sequence of stops on the ward round more efficient and decrease the spread of patients around the ward, making it less likely that someone is not seen. Hand trauma and breast surgery patients could be clustered in adjacent bays. However, this is practically very difficult with separate male and female bays and the need to juggle side rooms for those patients with diarrhea, methicillin-resistant *Staphylococcus aureus*, etc.

Bed utilization on the ward runs at a very high level (it is rare to see an empty bed) and patients are admitted on an ongoing basis to an available bed in a same-sex bay. It is also not clear when walking around the ward if the patient is a Plastic Surgery, Gynecology, or General Surgery patient. A clear printed card above the patient’s bed would make this far easier, or some sort of color coding. At present, the only way the people on the ward round know is to look at the list (was never printed for the start of the ward round) and rely on the house officer’s memory. This system is vulnerable if the house officer was to become sick, as they are the only doctor with that “panoramic” understanding of the patient’s on the ward. In addition, the list is also not typically structured in bay order, so the sequence of stops is not clear and again means reliance on house officer’s memory.

The consultation itself could be more standardized. Patients stated how they did not know who people were on the ward round, how they were not clear about plans and would have appreciated being given the opportunity to ask questions and check their understanding against that of the team’s.

#### Patient A

So like if we have questions, I don’t feel, I don’t feel comfortable being able to ask in front of everybody what I want to know because I feel stupid.

At the end of the ward round, a mini-checklist with the house officer would be useful. Do they have any questions? Are they clear about what jobs need to be done? What is the priority of the tasks? Are there potential problems that could be mitigated at this juncture? This can be run through with the list in hand. The same could be done with the nurses. For instance stating estimated discharge dates (if these are not already clear) so social care assessments and packages can be put in place in a timely manner.

### The role and significance of bottlenecks

A number of bottlenecks were identified. Some of the most significant and those that wasted the most person-minutes were:

1. Not having a nurse available for the ward round.
2. Needing to go and get gloves for each wound examination.
3. Patients dressings not down in time for the ward round.

The ward round takes about 50 minutes on average. With 3 nurses on the ward handling 23 patients (when at 100% bed utilization, which is very common) they are pushed for time. Nurses do not have a set “protected” amount of time set aside for the ward round since any spare time is taken up with nursing patients and the running of the ward. Doctors, however, do have till 9 AM when clinics start, to complete the ward round giving them about 35 to 40 minutes to complete it. The overrun to 50 minutes on an average means they are often late starting the morning clinic in outpatients (only once did the ward round finish within the allotted time). Hence the 3 nurses need to provide approximately 50 minutes of resource. But when plastic surgery, gynecology, and general surgery ward rounds come simultaneously, this resource is utilized 100% and there is no spare capacity for nursing patients, doing observations, taking patients to the toilet, rolling them, washing, feeding, dressing, changing bed sheets, answering phone calls from relatives, etc.

The demand on the nurses is then further increased when the Plastic Surgery ward splits into 2 or 3 to increase speed. This often means there is a delay in starting the ward round as the nurse has to finish the activity she/he had already started. The high variation shown in **Table** **1** further emphasizes the need for slack in the process. As the patient does not know exactly when the ward round is going to happen, they may often be in the toilet when the ward round comes (as occurred in ward round 3), meaning it has to double back or wait.

**Figure 12:**
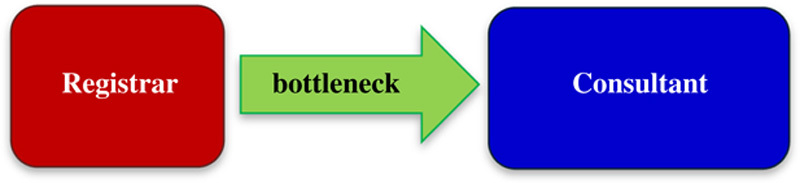
Current paradigm for decision-making on the ward round.

Another source of frustration identified by the nurses is the current paradigm for some of the decision making on the ward round (**Fig.** **12**).

**Figure 13:**
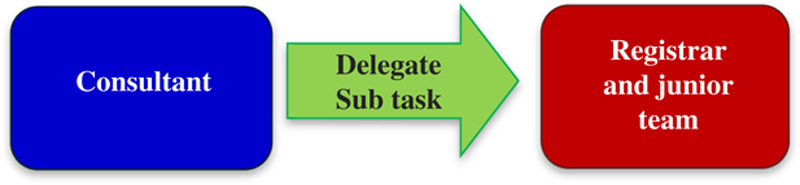
A possible alternative paradigm for decision-making.

The registrar will see a patient on the ward round and then have to go and speak to a consultant to determine the best course of action. This leads to the nursing staff and patient waiting for the registrar to come back later in the day with the plan. A better idea may be to have more consultant-led ward rounds, this may improve the training in decision making then as well (**Fig.** **13**).

Currently consultant-led ward rounds occur once per week with occasional ad hoc ward rounds for postoperative patients. However, the registrar led ward rounds are considered valuable in developing their decision making and leadership skills. It also allows them to develop their own style and interaction with other members of the team as well as the patient. Furthermore, consultants will not make decisions on each other’s patient’s, hence cannot lead the entire ward round.

Finally, if any of the resources identified in the IDEF0 map are missing, it can lead to a bottleneck, for example, going back to the computer to look up blood results, trying to find patient notes, etc.

### The key information flows, including the role of feedback

Information flow during the ward round can be variable. With so many people on the ward round (5 to 8), sometimes, people cannot physically fit around the bed or hear what is going on. Hence, their input may be limited.

Verbal information spoken by the patient and registrar needs to be translated into the notes as the formal record. However, the jobs that need to be done as a result of the consultation can be forgotten if they do not get written on a patient list, for example, ordering tests, etc. This may or may not be written down by the house officer or SHO. The registrar does not typically check in a systematic way that the house officer has acknowledged the jobs that need to be done and does not inspect the list where the jobs may be written. Ideally the SHO would do this since the house officer is often writing in the notes. The house officer’s understanding of the jobs that need to be done should be checked at the end of the ward round by an SHO who wrote down the jobs as they went along (this does not always happen).

The patient list itself, does lack a checklist, which ensures that certain information is captured. This could be amenable to modification. Feedback relates to the mechanisms that regulate the system and how it responds to change. These are poor. The main control is time, so ultimately the doctors need to move on and get through the ward round as a process. Hence, there is some urgency about the ward round. If a nurse is not available for the start of the round, the team will wait a short while (maybe a minute on average, while other people are also getting together) but will ultimately start and brief the nurse later. This prevents nursing input onto the round when its needed. Also, if a job created on the ward round was not done, members of the team will not be notified, it may be picked up later in the day by another member of the team, for example, another Registrar who did not lead the morning ward round. So there is little tracking of progress and information flow downstream.

### Motivation and incentives of system participants

The interviews and observations demonstrate that the house officers are motivated to impress their senior colleagues (being fresh out of medical school) and want the ward round and the ward generally to run smoothly since they work solely in that environment all day (unlike SHOs and Registrars who will be in clinic, other wards, operating theater, etc). House officers want clear plans so they can execute them efficiently.

#### Staff C—house officer

Amount of direction you get depends on the Registrar. You have to call the Registrar back to get discharge details like antibiotics and follow-up.

The Registrars want to make the right clinical decisions for the patients as a leader of the ward round and as a more senior responsible figure. The nursing staff would like the doctors to provide clear plans so their day can run more smoothly. They need to be clear since we know from the patient interviews how the nursing staff will provide much additional information and explanation to the patients after the doctors have gone (Appendix II, Supplemental Digital Content 1, http://links.lww.com/IJSO/A2 and Appendix III, Supplemental Digital Content 1, http://links.lww.com/IJSO/A2).

## Part B

Analyze the existing meta-processes that apply to the system for

- Adaptation to technological change
- Quality improvement

Meta-processes are smaller processes that lie behind or are associated with a larger one^18^. The meta-processes that apply to the system include:

- Needing a printer that functions and is well stocked with paper so that lists can be printed in time for the ward round.
- Black markers for the white board, which lists the patients, their locations on the ward, and their specialty or consultant.
- Availability of the operation note within the medical notes of postoperative patients.
- Not enough chairs, desk space, or available computers when they are needed most.
- Alcohol hand foam at the end of the patient’s bed.
- Adequate stock of medium-sized gloves.

Currently there is no system of checks to mitigate problems with the above list. There is no system to report to if a fault occurs. So no one checks at the start of the day if the printer has paper (it often does not), that the pens sitting at the bottom of the white board actually work and spares are available and people know where to get them from, that the operation note can be found as its been filed in the correct place, etc. When medium-sized gloves run out or alcohol hand foam is not available, there is no one to report the fault to there and then, no system to put in place a chain of events that rectifies this problem. Another adaptation could be to give the doctors their own belt-lipped alcohol dispenser to act as a backup.

The ward clerk is a key individual in making sure that administrative things are done and that excess stock is stored away neatly (eg, filing operation notes and enduring provision of paper to the printer).

## Part C

### Generate a costed and systematic plan for the improvement of the system, exploring improvements to both the substantive system and the meta-systems

Several quality improvement principles could be applied to the system, including use of lean and the Toyota Production system^19^, the need to decrease *muda* (Japanese for waste), and a focus on incentivizing the staff to do a job well and take pride in it as has been accomplished for example at the Shouldice clinic in Ontario, Canada^20^. These need to be integrated with the knowledge gained from the patient and staff interviews and the observational work. Ultimately a strategy which reduces steps in the process, reduces variation, and increases reliability and standardization while bringing a focus to patient-centered care would be the approach to take.

The substantive system could be improved from a staff and patient perspective by:

1. The ward round could potentially be standardized with a single registrar doing the ward round for the whole week. This would avoid multiple looks at dressings by different registrars, which disrupts and annoys the nurses. The same registrar would also be able to provide better continuity of care and get to the know patients and their clinical histories. This would require an adjustment of the rota but would not result in greater cost. Indeed, such a system exists at other hospitals but require careful planning.
2. Limit the number of people on the ward round to essential staff, making it more mobile, more private and intimate, less disruptive, and less intimidating for the patient.
3. A named nurse for the ward round who has been assigned that role for the day—their name will go onto the board and they should ensure they are available in time for the ward round (they cannot be sequestered by another specialty team, who would have their own dedicated ward round nurse). This will prevent the bottleneck of waiting for a nurse and will respect Little’s Law (work in progress=output rate×throughout time)^21^ and Queuing theory^22^.
4. There is no substitute for good communication and compassionate patient-centered care on the ward round. Examples include: getting down to eye level of the patient if practically possible, ensuring privacy (perhaps asking excess people to politely leave or not enter), checking patient understanding of the plan, giving them the opportunity to ask questions and encouraging their participation. Shared decision making is now a central theme of the Department of Health’s Liberating the NHS^23^ proposals encapsulated by the phrase: “No decision about me without me.”
5. Greater teaching, education, and understanding of the problems and potential solutions highlighted by this type of work should be fed back to staff in a multiprofessional setting through seminars, emails and posters, and reminders during handover (a guaranteed activity the staff will attend).
6. This work has given an insight into the Johari window^24^, for those leading the ward rounds and their team members. Recognition of how they are perceived by each other and by patients is an important part of reflective practice^25^.
7. Prioritizing the ward round as an important activity in the day that needs to be done well and in a more structured and systematic manner—like a nursing drug round or handover. It should be made part of professional development activities at the trust for the staff involved.
8. Improving the layout and design of the patient list. So important aspects related to quality of care are incorporated in a checklist like manner. Patients should be listed in bay order, not in the random manner they currently are. All of this is possible within the existing computer software, using existing functions, and the house officer investing more time in the updating of the list (5 min more per day).
9. If people see a problem in the system or with a meta-process, they can write it down on a *muda* board at the nurses desk (trialed as a simple clipboard that are abundant on the ward). Every day the *muda* board will be reviewed by nursing staff during handover, the ward clerk, and by doctors at the end of the ward round to see what faults and frustrations have been reported. The *muda* board would give visibility to problems, log them, and would also help generate ideas of how to deal with them through a proposed action column so people can see what has been suggested by others.
10. The findings of the *muda* board may need to be collated and ultimately presented at an audit half day so people can understand and take action collectively against consistently reported problems. A multidisciplinary, voluntary group of people from across administration, doctors, nurses, managers, pharmacists, etc., could take the lead in delivering practical solutions. This group could be called the *Kaizen* or improvement Committee, which would be backed by senior management and work locally on the ward but learn from other such groups around the hospital.

None of the above will cost in material terms, and extra time spent with the patient will free up house officer and nursing time later as the patient will not need them to explain what is going on. The *Kaizen* Committee can have a nominated quality improvement budget that would be set monthly by management after a trial and scoping exercise to determine the initial costs of this sort of activity.

There are some material costs, however:

- Buying a printer for the ward to ensure the list can be printed and the house officer does not have to go elsewhere (cost £99 approximately^26^).

The meta-processes could be improved by:

- Adjusting the ward clerk role to decrease *muda* on the ward but also making this more part of everyone’s role. The ward clerk would ensure that white board pens are available, gloves are in the racks, alcohol foam dispensers are at the end of every bed.
- Patients A and C remarked how having the doctor sit down at eye level would greatly help communication. Therefore, having a chair in the right place by the bedside would greatly help this. The house officer could potentially go around the ward before hand to ensure that these are in place as it will lead to better communication, patient satisfaction, and they will be called upon less to “fill in the blanks” after the other doctors have moved on.
- The costs of adjusting the ward clerk role would be an extra £4 an hour in salary (subject to negotiation). Hence, the approximate cost would be £4×8 hours per day×5 days a week×52 weeks a year=£8320. This would not include weekends when the ward clerk does not work, but ward rounds on the weekends are much smaller and lighter and there are less staff. The ward clerk being consistently onsite, could also be the person to whom failures could be reported to (via multiple channels, verbal, email, the *muda* board) and then rectified quickly and efficiently either in situ or through the *Kaizen* development committee. The additional money could fund extra hours of work or even a part time assistant.

**Table:**
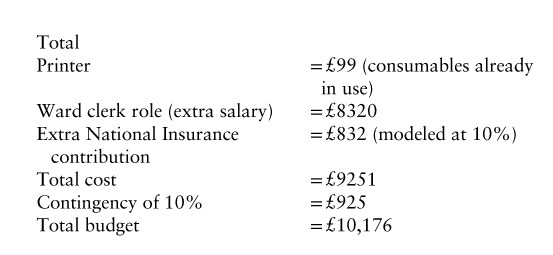


Some of these costs would be offset by the 31 person-minutes on average wasted in each daily ward round. Modeling a salary of £20 per hour (pretax) for the 6 or so members=£10/day saved=£10×5 days×52 weeks=£2600 saved.

Net investment would thus be: £10,176−£2,600=£7576.

One cannot put a price on increased patient satisfaction with the ward round but this is something to have in the equation. The Care Quality Commission’s NHS Inpatient survey revealed how important communication is to patients; specifically answering questions, talking as if they are not there, appearance of teamwork, availability of staff, and involvement in their own decision making^27^.

Progress would be tracked through Deming’s PDCA cycles and statistical process control charts for key target areas^28^. In time, as ward efficiency increases it is conceivable that patient safety could improve, resulting in less avoidable harm. For instance, thromboprophylaxis rates could increase resulting in lower rates of deep vein thrombosis and pulmonary embolism. This would also result in higher direct incomes for the trust through the Commissioning for Quality and Innovation (CQUIN) payment framework mechanism (of which venous thromboembolism is a part)^29^.

### Limitations

The limitations of this work include how different professionals and patients may have their own biased viewpoint and the small sample sizes are not representative in a statistical way. The ward on which the work was done had only been in use for a short while since it opened in early November and hence there may be an element of bedding down.

### Further work

This work will be extended further by examining additional data sources such as incident reports and patient complaints. Potentially other patient groups like children and their parents as well as burn patients could be involved to determine the special needs of these patient subgroups. The results of this study will be fed back to the department of Plastic Surgery and the Clinical Audit department and will be presented to staff at an audit half-day meeting. The patients who participated in this work will also be informed of its progress through periodic updates from the author.

## Conclusions

This small-scale study demonstrates how the ward round as a process can be assessed, including product quality, process quality, the measurement and management of capacity, the role of standardization, the role and significance of bottlenecks, the key information flows, including the role of feedback, and the motivation and incentives of system participants and ideas for improvement generated.

## Supplementary Material

Supplemental Digital Content is available for this article. Direct URL citations appear in the printed text and are provided in the HTML and PDF versions of this article on the journal's Website, www.IJSOncology.com.
